# Nerve injury and repair in a ketogenic milieu: A systematic review of traumatic injuries to the spinal cord and peripheral nervous tissue

**DOI:** 10.1371/journal.pone.0244244

**Published:** 2021-01-04

**Authors:** Jamasb Joshua Sayadi, Lohrasb Sayadi, Ellen Satteson, Mustafa Chopan

**Affiliations:** 1 Stanford University School of Medicine, Stanford, California, United States of America; 2 Department of Plastic Surgery, University of California, Irvine, California, United States of America; 3 Division of Plastic and Reconstructive Surgery, Department of Surgery, University of Florida Health, Gainesville, Florida, United States of America; "INSERM", FRANCE

## Abstract

Dietary interventions such as intermittent fasting and the ketogenic diet have demonstrated neuroprotective effects in various models of neurological insult. However, there has been a lack of evaluation of these interventions from a surgical perspective despite their potential to augment reparative processes that occur following nerve injury. Thus, we sought to analyze the effects of these dietary regimens on nerve regeneration and repair by critical appraisal of the literature. Following PRISMA guidelines, a systematic review was performed to identify studies published between 1950 and 2020 that examined the impact of either the ketogenic diet or intermittent fasting on traumatic injuries to the spinal cord or peripheral nerves. Study characteristics and outcomes were analyzed for each included article. A total of 1,890 articles were reviewed, of which 11 studies met inclusion criteria. Each of these articles was then assessed based on a variety of qualitative parameters, including type of injury, diet composition, timing, duration, and outcome. In total, seven articles examined the ketogenic diet, while four examined intermittent fasting. Only three studies examined peripheral nerves. Neuroprotective effects manifested as either improved histological or functional benefits in most of the included studies. Overall, we conclude that intermittent fasting and the ketogenic diet may promote neuroprotection and facilitate the regeneration and repair of nerve fibers following injury; however, lack of consistency between the studies in terms of animal models, diet compositions, and timing of dietary interventions preclude synthesis of their outcomes as a whole.

## Introduction

Wound healing is a biological phenomenon that requires sufficient calories and precursors for unimpeded progression [1, 2]. The association of malnourishment and adverse healing serves as the basis for nutritional optimization, and surgical emphasis is placed on timely recognition of deficiencies and appropriate supplementation [3]. Yet, emerging evidence suggests that diet can also be leveraged to augment the reparative process [4–7]. Intermittent episodes of energy restriction (i.e. intermittent fasting) and regimens that promote metabolic switching—namely, ketone oxidation—have shown salutary effects across diverse species and diseases [8–19]. These dietary interventions seem to induce novel responses to injury and enhance repair in various tissues [20–26]. Interestingly, the proposed mechanisms extend beyond weight loss or diminished production of reactive oxygen species and include adaptive stress responses, reduced inflammation, efficient bioenergetics, and improved repair and renewal at the cellular level [6–7]. Ketone bodies—by-products of fatty acid oxidation and a vital fuel source for the brain in episodes of nutrient deprivation—have been implicated as key mediators [27].

Amongst nutritional regimens that mimic fasting-state metabolism, the ketogenic diet (KD) is well studied [28]. This diet relies primarily on the oxidation of lipids and was discovered as a treatment for drug-resistant epilepsy over a century ago. Since then, KD has demonstrated protective effects in a wide-spectrum of neurological disorders, including models for neurodegeneration, cerebrovascular disease, and immune-mediated demyelination [27–33]. The diet is also relatively simple to follow in practice, with patients instructed to consume meals containing an average of four parts fat to one part protein and carbohydrates (4:1 lipid to nonlipid ratio) such that 90% of daily caloric intake is derived from fat [34, 35]. A modified form of KD with a 3:1 ratio of lipid to nonlipid can also be used [35, 36]. Working with a nutritionist, patients can design a ketogenic diet by strategically avoiding excessive intake of foods such as processed grains, fruits, and beans while consuming more calories through foods such as eggs, meat, dairy, and nuts.

In addition to KD, intermittent fasting is another dietary intervention that has been well studied in the literature. For instance, every-other-day fasting (EODF), also known as alternate day fasting (ADF), is a form of intermittent fasting in which patients are instructed to alternate days between zero caloric intake and *ad libitum* food consumption. In turn, EODF has been shown to significantly increase serum ketone body levels in animal models, while a randomized controlled trial of EODF over four weeks in humans has even demonstrated improved cardiovascular markers, improved fat-to-lean ratios, and increased serum ketone bodies in subjects, even on non-fasting days [37–39].

Despite the promising research surrounding KD and intermittent fasting, there remains a paucity of surgical interest surrounding these dietary interventions [40–46]. The potential for neuroprotection and enhanced reparative processes make KD and related diets attractive adjuncts in peripheral nerve surgery and optimizing outcomes. Thus, we set out to investigate the effects of KD, intermittent fasting and ketone bodies on nerve injury and repair. To this effect, we performed a systematic review of the available literature using various keywords and inclusion criteria, with emphasis on traumatic injuries of the spinal cord and peripheral nerves.

## Methods

In accordance with PRISMA (Preferred Reporting Items for Systematic Reviews and Meta-Analyses) guidelines, two reviewers (MC, LRS) conducted a systematic literature search within the MEDLINE database. All articles published between 1950 and 2020, with English as the primary language, were included in the search query. A combination of Boolean operators with the following MeSH terms were used to conduct the search: “wound healing,” “cellular repair,” “regeneration,” “tissue healing,” “stem cells,” “tissue repair,” “nerve injury”, “nerve repair,” “nerve regeneration,” “spinal cord injury,” “ketogenic diet,” “ketosis,” “ketone body,” and “ketones.” Both reviewers independently performed each step in the study selection process. Cross-referencing initial articles allowed for the identification of additional articles. Articles were screened by title and available abstract for nerve repair and regeneration involving the peripheral or spinous tissues. The optic system was excluded from this review. Injuries that were traumatic or surgical in nature were included, whereas ischemic, neurodegenerative, immunologic, neoplastic, epileptic and metabolic insults were excluded. Studies that employed a ketogenic diet or ketone supplementation as an intervention were included for further review. Diets that mimicked a fasting state and promoted ketosis (i.e. very low carbohydrate diet, intermittent fasting) were also included. Studies that dealt with synthetic ketone analogues (i.e. poly-3-hydroxybutyrate), review articles, book chapters, editorials, and commentaries were excluded. Finally, studies that did not examine a histological or functional outcome were excluded.

### Data extraction

Both reviewers independently verified that all studies included in systematic review met inclusion criteria and omitted exclusion criteria. The manuscripts were then analyzed with respect to study subjects, interventions, and findings. Studies were classified based on their methodology and their use of control or comparative cohorts. Studies were further scrutinized for their intervention, with respect to the dietary regimen, ketone body supplementation, duration, and measurement of serum ketones. Finally, the studies’ main findings were analyzed and succinctly presented in a summative table.

## Results

**Fig 1:**
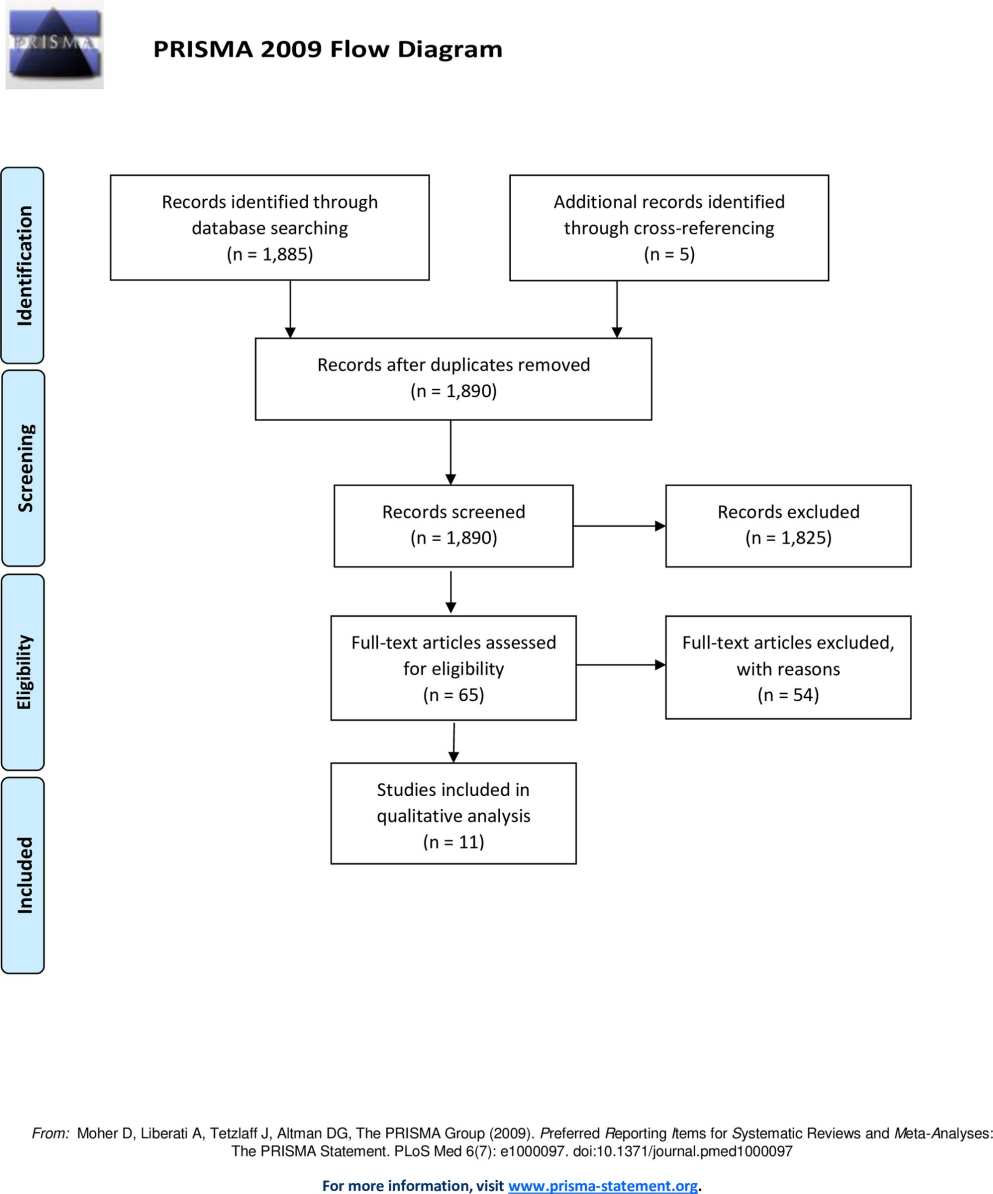
Flow diagram. A total of 1890 articles were obtained through database searching and cross-referencing. After reviewing all 1890 abstracts, only 65 articles met the inclusion criteria and moved onto full-text assessment. Following full-text assessment, only 11 articles met the selection criteria for further qualitative analysis.

Systematic search of the MEDLINE database yielded 1,885 articles. After screening and cross-referencing for an additional five citations, 65 articles were reviewed in full-text, and 11 articles were deemed eligible for inclusion in the analysis. A flow diagram is depicted in Fig 1. The included studies primarily concerned the spinal cord with only three studies involving peripheral nerves. All studies were designed with control groups and/or comparative cohort(s) utilizing rats as subjects, except for two studies which utilized mice (Streijger et al., 2011 and Mayr et al., 2020) [47, 48]. One study (Mayr et al., 2020) investigated impact of KD in the setting of both central and peripheral nervous system injury [48]. A summative review of the included articles is provided in Table 1.

**Table 1:** Summary of articles. Articles included in the qualitative analysis were organized by type of nervous system injury, dietary intervention, timing of diet initiation relative to injury, duration of dietary intervention, whether or not serum ketones were measured, and brief summary of results.

Article	Nervous System Injury	Dietary Intervention	Timing	Duration	Serum Ketones Tested	Outcome
Tan 2020	C5 spinal cord hemi-contusion	KD (3:1 ratio of fat to carbohydrates plus protein) + additional 1000–1500 mg βHB BID for first four days post-injury	Post-injury	8 weeks	Yes	• Increased neuronal and axonal sparing in dorsal cortico-spinal tract
						• Improved forelimb functional recovery
Mayr 2020	Tibial and peroneal nerve transection; T10-T11 spinal cord hemi-contusion	KD (2.8:1 ratio of fat to carbohydrates plus protein)	Pre- and Post-injury	1 week pre-injury, 4 weeks post-injury	Yes	• No effect
Li 2020	Sciatic nerve crush injury	KD (3:1 ratio of fat to carbohydrates plus protein)	Post-injury	8 weeks	Yes	• Increased total axons, axon diameter and density, axon/fiber ratio and myelin thickness
						• Improved nerve regeneration and functional recovery
Lu 2018	C7 spinal cord hemi-contusion	KD (4:1 ratio of fat to carbohydrates plus protein)	Post-injury	4 weeks	Yes	• Diminished oxidative stress and inflammation (↓ blood myeloperoxidase, ↑ blood superoxide dismutase)
						• ↓NF-κB signaling, ↓TNF-α, ↓IL-1β, ↓IFN-γ, ↑Nrf2 in injured spinal cord
						• Improved forelimb functional recovery
Liskiewicz 2016	Sciatic nerve crush injury	KD (3.4:1 ratio of fat to carbohydrates plus protein)	Pre- and/or post-injury	3 weeks pre-injury, 6 weeks post-injury	Yes	• Improved myelin thickness, axon diameter, fiber diameter, axon/fiber diameter ratio, myelin thickness/axon diameter ratio, circularity
						• Neuroprotective in pre-conditioned subjects
Streijger 2014	C5 spinal cord hemi-contusion	KD (3:1 ratio of fat to carbohydrates plus protein)	Post-injury	10 weeks	No	• No effect
Streijger 2013	C5 spinal cord hemi-contusion	KD (3:1 ratio of fat-to-carbohydrate plus protein)	Post-injury	12 weeks	Yes	• Reduced lesion size and gray matter sparing
						• Neuroprotective and enhanced functional recovery
Streijger 2011	T10-T11 spinal cord contusion	Every-other-day fasting	Post-injury	14 weeks	Yes	• No effect
Jeong 2011	T10 spinal cord contusion	Every-other-day fasting	Pre- and/or Post-injury	3 weeks pre-injury, 10 weeks post-injury	No	• Enhanced functional recovery in pre- and post-injury groups
Plunet 2010	C4 spinal cord hemi-contusion	Every-other-day fasting	Pre-injury	6 weeks	No	• Increased number of undamaged neurons, gray matter sparing and reduced lesion size
						• Neuroprotective and enhanced functional recovery
Plunet 2008	C4 spinal cord hemi-contusion	Every-other-day fasting	Post-injury	33 days	Yes	• Reduced lesion size, increased corticospinal tract plasticity and gray matter sparing
						• Neuroprotective and enhanced functional recovery

### Peripheral nervous system

Three studies examined the impact of KD on peripheral nerve regeneration. In the first study, Li et al. evaluated the effects of KD with and without concurrent electrical stimulation on neuromuscular recovery in rats [49]. Four hours after sciatic nerve crush, rats were assigned to either a regular carbohydrate-based diet or KD characterized by a 3:1 ratio of fat to carbohydrates and protein. Electrical nerve stimulation (2Hz, 1mA) was also performed on some rats within both dietary groups, causing contraction in two muscles innervated by the sciatic nerve (gluteus maximus, biceps femoris) for 15 minutes every other day. These interventions were performed for a total of 8 weeks following nerve injury. In turn, the researchers found that the biceps femoris produced a more robust electromyography (EMG) signal in rats receiving KD alone relative to controls and an even greater signal when KD was combined with electric stimulation relative to KD alone. However, the gluteus maximus produced a stronger EMG signal in solely the KD with electrical stimulation group relative to controls and KD alone. On histology, rats fed KD alone had increased mean myelin thickness and axon/fiber diameter compared to those of controls. These findings were significantly greater in rats receiving the ketogenic diet and electrical stimulation. Finally, Li et al. also reported improved functional recovery in rats fed KD relative to controls based on sciatic functional index scores, which were once again greater in rats fed KD with electrical stimulation relative to KD alone.

In the second study, Liskiewicz et al. also investigated the impact of KD on sciatic nerve regeneration in rats [50]. The researchers subjected rats to sciatic nerve crush injury and subsequently assigned the animals to KD (79% fat, 9.5% protein, 0.8% carbohydrates) or a standard high-carbohydrate diet. A third arm was also included in which rats were preconditioned with KD for 3 weeks prior to nerve crush. Rats in all three groups were then maintained on their respective diets for 6 weeks following injury. After this time period, the researchers found that regenerating nerves in the preconditioned KD group were most similar to those of uninjured rats based on a variety of histomorphometrical parameters, including myelin thickness, fiber density, and fiber diameter. However, functional recovery in neither the preconditioned nor the post-injury KD groups showed significant differences relative to rats receiving standard diet at any time point based on CatWalk gait analysis, a validated functional test for gait in rats, with all three groups showing recovery to near-baseline gait after only 3 weeks.

In the third study, Mayr et al. examined effects of KD on sensorimotor recovery from complete transection of the common peroneal and tibial nerves [48]. Mice were fed either a standard or ketogenic diet (8.6% protein, 75.1% fat, and 3.2% carbohydrates) beginning 7 days before nerve transection and continued up to 28 days following the injury. Ketone levels were significantly elevated in the serum of mice fed KD relative to those fed standard diet on the day of spared nerve injury (SNI). The researchers subsequently reported no significant differences at 28 days following SNI between the two dietary groups on a variety of motor function tests, including total distance walked over thirty minutes during open field testing and hindlimb function during ladder rung testing in which mice were subjected to walking across a horizontal ladder. Moreover, the researchers also found no difference at 28 days following SNI in terms of sensory recovery from mechanical allodynia in mice fed KD and standard diet, as measured by a nociception test known as the von Frey assay.

### Spinal cord

Nine articles examined the impact of either KD or every-other-day fasting (EODF), a common variant of intermittent fasting, on recovery following surgically induced spinal cord injuries (SCIs). Three of the nine studies showed no benefit from either dietary intervention. In the first of the non-supportive studies, Streijger et al. (2014) subjected rats to C5 SCI before assigning them to either a standard carbohydrate diet or KD consisting of a 3:1 ratio of fat to carbohydrates plus protein beginning four hours after cervical injury for a total of 1 week [51]. Rats in the KD group were also administered other agents, including ghrelin, ibuprofen, and C16 (an anti-apoptotic agent), previously reported to confer benefits for spinal cord recovery. These compounds were given between hours to days after the injury, for varying time periods, and through a variety of routes (e.g. oral, intravenous, intraperitoneal). In turn, the researchers reported significant functional improvement on the Montoya staircase test, a validated behavioral test of forelimb function in rats, in the combinatorial group relative to controls at 10 weeks post-injury. However, no significant differences were reported between combinatorial and control groups on other measures of functional recovery, including the rearing test, grooming test, or horizontal ladder test. Similarly, histological analysis revealed no significant differences between the two groups in terms of white or gray matter sparing at any spinal cord level. The study’s authors concluded that these findings suggest potential interactions between the agents and ketogenic diet used in the combinatorial treatment that may have negated the beneficial effects previously reported for each individual intervention in the setting of SCI.

The second non-supportive study was conducted by a similar group of investigators led by Streijger et al. (2011) [47]. Following surgically induced thoracic SCI (T10-T11 contusion), C57BL/6 transgenic mice were assigned to receive a standard diet (28.5% calories from protein, 13.5% from fat, and 58.0% from carbohydrates) either *ad libitum* (AD) or every other day with the first 24 hours of food deprivation beginning immediately after injury. The mice were maintained on these diet regimens for 14 weeks thereafter, during which time the researchers observed no significant differences between the groups in terms of hindlimb motor function recovery or gray/white matter sparing. The investigators noted that EODF mice exhibited no significant increase in their serum ketone bodies relative to pre-op levels by the end of their second, third, and fourth days of fasting in contrast to rats subjected to the same diet.

In the final non-supportive study, Mayr et al. studied effects of KD on sensorimotor recovery from surgically induced thoracic hemisection over the T10-T11 vertebrae [48]. Mice were fed either a standard or ketogenic diet beginning 7 days before SCI and continued up to 28 days following the injury. The KD was composed of 8.6% protein, 75.1% fat, and 3.2% carbohydrates, and blood ketone concentrations were confirmed to be significantly elevated in KD mice prior to SCI. In turn, the researchers reported no significant differences at 28 days following SCI between the two dietary groups on a variety of motor function tests, including total distance walked over thirty minutes during open field testing and hindlimb function during ladder rung testing in which mice were subjected to walking across a horizontal ladder. Moreover, the researchers also found no difference at 28 days following SCI in terms of sensory recovery from mechanical allodynia in mice fed KD and standard diet, as measured by von Frey assay.

In contrast, the remaining six studies of the central nervous system were decisively supportive. Using rats subjected to C5 hemicontusion injury, Streijger et al. (2013) investigated the effects of KD characterized by 3:1 ratio of fat-to-carbohydrate plus protein initiated four hours post-injury [52]. Relative to rats fed a standard carbohydrate-based diet, KD rats displayed reduced lesion size and sparing of gray matter on histology that correlated improved forelimb use and behavioral recovery after 14 weeks of KD. Moreover, serum ketones were confirmed to be significantly elevated in the KD group relative to standard diet beginning one day after injury. KD also significantly increased expression of glucose transporter-1 (GLUT1) and monocarboxylate transporter-1 (MCT1) on histological analysis of the lesioned spinal cord. Pharmacological inhibition of MCT1 with intrathecally-administered 4-CIN yielded no improvements in lesion size in KD rats relative to SD rats treated with the same compound, which the authors concluded further supported a causative role for ketogenesis in functional recovery after SCI.

Tan et al. led another study investigating whether ketogenic diet supplemented with exogenous ketones could improve histological and functional outcomes in Sprague-Dawley rats subjected to C5 spinal cord hemi-contusion injury [53]. The experimental group was specifically fed a ketogenic diet comprised of a 3:1 ratio of fats to carbohydrates plus protein beginning 3 hours post-injury. The rats were also given ketogenic salt gavages containing 1000–1500 mg β-hydroxybutyrate every 12 hours for four days post-injury. Blood ketones were found to be significantly elevated in rats the ketogenic diet with ketone supplementation (KS) relative to control rats fed a standard diet by one day after the C5 hemi-contusion. Interestingly, oral ketone salt supplementation with KD did not appear to significantly elevate blood ketone levels higher than KD alone at this time point. The authors ultimately found that KD with KS improved forelimb motor recovery as measured by performance on the Montoya staircase test at 6 weeks post-injury. Furthermore, they also found increased neuronal sparing 2.4 mm rostral to the lesion epicenter as well as increased axonal density in the dorsal corticospinal tract in KD with KS rats relative to controls.

The third study examining ketogenic diet and spinal cord injury was led by Lu et al. [54]. The researchers fed Sprague-Dawley rats either a standard diet or ketogenic diet with a 4:1 ratio of fat to carbohydrate and protein beginning four hours after C7 spinal cord hemi-contusion. β-hydroxybutyrate were found to be significantly elevated in KD rats relative to controls as early as one day after injury and for the remaining four weeks of dietary intervention. In turn, the researchers reported that KD rats experienced greater motor recovery as measured by the cylinder rearing test and a modified Montaya staircase test by three and four weeks after injury, respectively. Lu et al. also ran several additional experiments to help elucidate potential mechanisms behind their behavioral findings. In particular, they measured greater levels of the anti-oxidative marker superoxide dismutase as well as lower levels of the inflammatory marker myeloperoxidase in the blood of KD rats 4 weeks post-injury. Additionally, the authors found significantly elevated levels of Nrf2 (a regulator of the oxidative stress response) and reduced pro-inflammatory mediators (TNF-α, IL-1β, IFN-γ, NF-κB pathway) within the spinal cord tissue of KD rats 4 weeks post-injury. They conclude that results provide potential mechanisms behind improved functional recovery in KD rats following SCI.

The remaining three studies examined the impact of intermittent fasting on SCI recovery. Jeong et al., for example, looked at the effects of EODF on recovery from T10 contusion injury in rats [55]. The study included an *ad libitum group*, a post-EODF group that started their first fast immediately following injury, and a pre-EODF group that started their first fast 3 weeks prior to injury. Each diet was then maintained for three weeks following thoracic SCI. Relative to the AL group, both EODF groups exhibited better functional recovery based on Catwalk gait analysis and the Basso-Beattie-Bresnahan (BBB) ambulatory assessment, another validated test for locomotor function in rats; however, no significant group differences were found on histological analysis of gray and white matter sparing. In a similar study, Plunet et al. (2010) investigated the impact of EODF initiated one month prior to C4 SCI and maintained for 6 weeks post-injury in rats [56]. Compared to controls fed AL, EODF rats experienced significantly better forelimb functional recovery, reduced lesion size, and increased numbers of undamaged neurons on histology. In the third study, a similar group of investigators led by Plunet et al. (2008) examined the effects of EODF when initiated after C4 SCI and continued for 3 weeks post-injury [57]. Rats fed with an EODF regimen once again displayed better gait pattern, forelimb function, and vertical exploration, as well as dramatically reduced lesion sizes on histology relative to AL controls. Moreover, EODF rats also displayed greater corticospinal sprouting both rostral and caudal to the lesioned spinal cord, suggesting enhanced neuronal plasticity. The investigators also measured β-hydroxybutyrate levels to be elevated in the serum of EODF rats on fasting days compared to that of controls.

## Discussion

Outcomes in peripheral nerve surgery are influenced by numerous clinical elements which are thoughtfully considered when formulating treatments [58]. Such factors consist of the mechanism and extent of injury, timing, length of nerve gaps, material used to bridge gaps, quality of donor nerves and end organs, the surrounding wound bed, coaptive techniques, and post-operative rehabilitation. While advancements in recent decades have broadened our therapeutic armamentarium with innovative surgical approaches, including nerve transfers, targeted muscle reinnervation, and regenerative peripheral nerve interfaces [59–61], there are certain factors that curb craft and technique. The reparative capabilities of the host are a key bottleneck in healing, and as suggested by this systematic review, the ketogenic diet and similar dietary interventions may facilitate this process.

**Fig 2:**
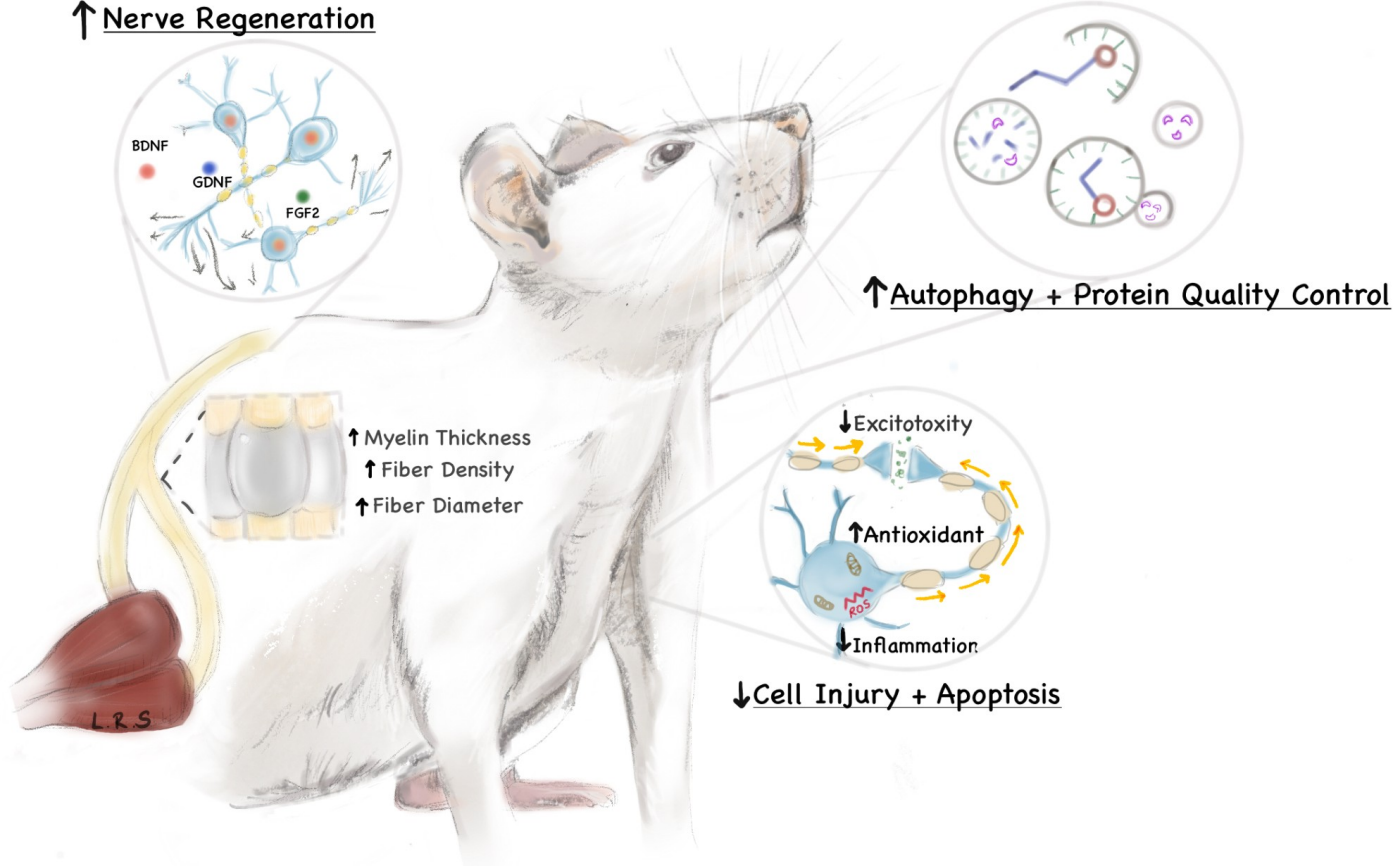
Potential mechanisms promoting nerve regeneration and repair in a ketogenic milieu. The ketogenic diet may promote regeneration and repair following neuronal injury through a variety of mechanisms, including upregulation of neurotrophic factors, enhanced autophagy, diminished excitotoxicity, and reduced inflammation. The mediators of these effects are thought to be ketone bodies, which are hepatically-derived byproducts of fatty acid oxidation formed during fasting or low-carbohydrate states.

The potential mechanisms are likely multifaceted (Fig 2), yet the preponderance of pre-clinical and clinical data suggest a protective quality to these dietary interventions [7, 8, 27, 30, 62–64]. Diminished excitotoxicity, reduced inflammation and heightened antioxidant capacities may limit the extent of cell injury, death and apoptosis [26, 65, 66]. Enhanced autophagy and protein-quality measures may also contribute to improved cellular survival or elimination of degrading neural elements [22, 67, 68]. Furthermore, there is an upregulation of neurotrophic factors (such as BDNF, GDNF, FGF2) which may augment the elongation and maturation of regenerating nerve fibers [21, 69]. Additional mechanisms may include inhibition of histone deacetylase (HDAC) activity, NLRP3 inflammasome activity, NF-κB signaling, myeloperoxidase activity, and pro-inflammatory cytokine TNF-α, IL-1β, IFN-γ signaling [54, 70, 71]. Increased activity of the anti-oxidative response regulator Nrf2 as well as superoxide dismutase have also been posited to play a mechanistic role in improved neural recovery from injury [54]. Neuroprotection is afforded in nearly all models of neurological insult, including ischemic, immune-mediated, degenerative, and traumatic injuries. This is further supported by the observation that ketogenic regimens may improve the frequency and intensity of migraines [72]—a complex neurologic disorder with both central and peripheral potentiators. While the reparative processes in the central and peripheral nervous system are distinct, the evidence provided in this systematic review suggests a protective effect in both systems.

Interestingly, the mediators of the beneficial effects are thought to be ketone bodies, which are hepatically-produced from fatty acid oxidation and serve as vital alternative metabolic sources during fasting times. Ketone bodies are pleotropic and may exert their influence through non-canonical mechanisms, such as epigenetic modification and G-coupled receptor signaling [73, 74]. Such methods induce the expression of genetic loci responsive to BDNF and oxidative stress and reduce inflammation by diminishing the production of pro-inflammatory cytokines and translocation of NF- κB [75, 76]. While negative energy balance and weight loss are valid concerns of these dietary regimens, the caloric intake may be improved given the fuel-efficiency of lipids and ketones [33–35]. Additionally, improvements in cellular enginery (i.e. mitochondrial biogenesis) may improve overall bioenergetics and lead to accelerated wound healing [77–79]. This is suggested by animal studies which observed quickened cutaneous and intestinal healing times in burns and colonic anastomoses, respectively [80, 81]. Altogether, the multidimensional role of ketone bodies likely developed to combat the fundamental challenge of nutritional deprivation during our evolution, with robust homeostatic processes in the brain and other organ systems that are designed optimize performance and resistance to disease and injury. Dietary regimens that induce or mimic the fasting state may capitalize on this adaptive machinery.

Given the non-invasiveness of these dietary interventions and the encouraging results witnessed in patients with acute cerebral or spinal cord disease, a logical extension would be to apply them in the context of surgical management of peripheral nerve injuries and reconstructions. Lending further credibility to this notion is the well-documented observation that neurons in the CNS have a limited ability to regenerate their axons following injury, whereas neurons in the PNS typically have greater regenerative potential [82]. Yet, no clinical trials to date have studied the impact of KD on recovery from peripheral nerve injuries, while one randomized clinical trial led by Yarar-Fisher and colleagues is currently recruiting participants to help assess the safety and efficacy of KD in improving motor and sensory function following acute cervical or thoracic spinal cord injury (ClinicalTrials.gov Identifier: NCT03509571) [83].

Our systematic review identified two studies that suggest a ketogenic diet may confer significant benefits in the setting of peripheral nerve injury, though these studies also share important differences that should be taken into consideration when comparing their results [48, 49]. For instance, the ketogenic diet composition was different in each study, with Liskiewicz et al. using a KD formulation that contained a greater ratio of fat to carbohydrates. The researchers in both studies addressed potential concerns about their respective KDs by measuring serum β-hydroxybutyrate levels before and during their dietary interventions. In turn, they ensured that KD groups were in a state of ketogenesis while controls were not, which was the intention of their ketogenic diets regardless of their specific nutrient breakdowns. Furthermore, Li et al. included electrical stimulation in their study and consequently identified a synergistic effect of combined KD and stimulation on both histological and functional recovery. In contrast, Liskiewicz et al. added a KD preconditioning arm to their study and in turn identified benefits within only this group upon histological analysis. Despite these differences, the two studies illustrate that KD may confer some benefits in the setting of peripheral nerve injury in rats, though the optimal timing of diet relative to injury, duration of diet, and functional impact remain unclear based on their results.

The efficacy of the ketogenic diet and intermittent fasting has been demonstrated in the acute setting, yet a prophylactic benefit may also be derived, particularly from caloric restriction. This is otherwise known as “hormesis,” in which exposure to mild stress prompts adaptive responses that may protect cells and organs from severe forms of stress and injury [6]. As such, the preconditioning effect of intermittent fasting may be rendered preoperatively, in efforts to optimize outcomes in delayed or elective nerve reconstructions and transfers. Patient compliance likely poses the greatest obstacle in their assimilation into prehabilitative or rehabilitative programs. The notion of three meals per day interspersed with snacks is ingrained in our culture and society, making time-restricted feeding a difficult task to fulfill. Contrastingly, the ketogenic diet is well tolerated and may be better suited for our lifestyle needs. Serum ketone testing may also provide us with a regulatory measure to confirm dietary adherence. Not all tissues, however, may benefit from these dietary regimens, particularly bone tissue with concern for resorption and increased catabolism under a ketogenic diet [84, 85]. Nevertheless, the behavioral basis of these interventions, along with its unfamiliarity amongst patients and providers, may require the expertise and services of nutritionists to implement these dietary regimens safely and adequately.

Of note, three studies analyzed in this meta-analysis did not report beneficial findings for ketogenic diet or intermittent fasting in the setting of CNS/PNS injuries. The first of these studies was Streijger et al. 2011, which utilized a mouse model (C57BL/6) that did not have significantly elevated serum ketone bodies on the second, third, and fourth days of intermittent fasting relative to Sprague-Dawley rats subjected to the same diet [47]. It is possible that these mice were exhibiting an intrinsically attenuated ketogenic response to fasting, which may have contributed to the non-supportive findings. The second study was conducted by a similar group of authors (Streijger et al. 2014), this time using Sprague-Dawley rats [51]. However, the rats were administered ghrelin, ibuprofen, and C16 in addition to being placed on KD. This combinatorial treatment may have negated any beneficial effects conferred by ketogenic diet, and the authors did not measure serum ketone levels in the rats at any point. Finally, the last non-supportive study was conducted by Mayr et al. [48], who used the same mouse model as the one in Streijger et al. (2011) to examine the impact of KD on sensorimotor recovery after spinal cord and peripheral nerve injuries. In addition to the potentially attenuated response of the C57BL/6 mice to ketogenic diet, a significant limitation of this study was the fact that these mice were subsequently maintained on their diet for only 4 weeks post-injury, which may have been insufficient time to witness the any functional improvement.

This systematic review is not without limitations. While our analysis argues for a favorable effect of ketogenic diet and intermittent fasting on nerve injury and repair, the evidence is only suggestive as there is a paucity of rigorous clinical data. Similarly, there was a lack of consistency between many of the studies in terms of animal model, precise formulation of the diets, and timing of dietary interventions. These differences contributed to heterogeneity between the studies, precluding the synthesis of outcomes and comparison of ketogenic diet with intermittent fasting. Additional limitations include the possibility that all articles related to this topic were not included in our analysis as only one database was utilized for this investigation. Nonetheless, the broad search criteria allowed for large-volume screening of the available literature and was strengthened by cross-referencing. The focus of this systematic review was refined to the nervous tissues and injurious mechanisms that are most encountered by reconstructive surgeons in the clinical setting, and as such, articles involving the optic and cerebral nervous systems were not included. Of the included studies, several were attributed to similar groups of authors (e.g. Streijger et al. and Plunet et al.) and thus cannot be considered strictly independent from one another. Lastly, inter-rater reliability for study selection was not statistically analyzed.

## Conclusions

Dietary interventions may be leveraged to facilitate the healing process in the spinal cord and peripheral nervous system. Diets that promote a ketogenic milieu may enhance resistance to nerve injury and regeneration. Further research should aim to investigate these effects in clinical settings.

## Supporting information

S1 Checklist PRISMA 2009 checklist. (TIF)
