# Supplementary figures and images for: Nerve injury and repair in a ketogenic milieu: A systematic review of traumatic injuries to the spinal cord and peripheral nervous tissue
Source: PLoS One. 2021 Jan 4;16(1):e0244244. doi: 10.1371/journal.pone.0244244 (PMC7781473; doi:10.1371/journal.pone.0244244)

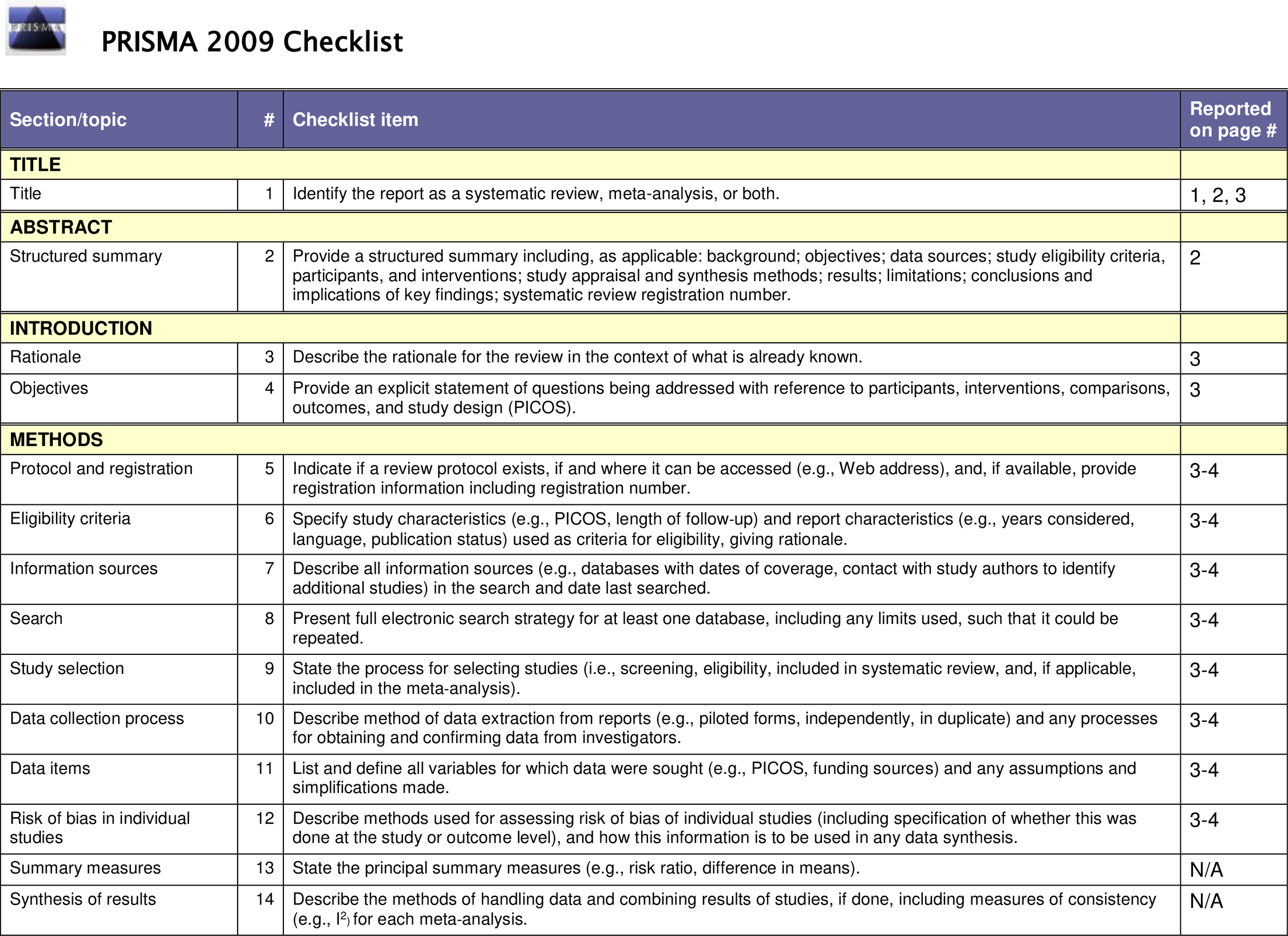

Supplement: S1 Checklist — (TIF) [file pone.0244244.s001.tif]
